# Supplementary material for: Analysis of unusual and signature APOBEC-mutations in HIV-1 pol next-generation sequences
Source: PLoS One. 2020 Feb 26;15(2):e0225352. doi: 10.1371/journal.pone.0225352 (PMC7043932; doi:10.1371/journal.pone.0225352)
Supplement: S1 Text — (DOCX) [file pone.0225352.s001.docx]

Supplementary Text

In a previous study, we used a mixture model and showed that the presence of ≥3 signature APOBEC mutations predicted risks of hypermutation of 99.8%, 98.5%, and 97.8% in PR, RT, and IN, respectively. However, since that study was published, we updated the list of signature APOBEC mutations. Additionally, in the current study, we applied the cutoff of ≥3 signature APOBEC mutations to whatever part of the *pol* gene that was sequenced whether it was PR+RT, PR+RT+IN, or IN alone (Table 1). As a result of these changes we performed an additional analysis to evaluate the use of ≥3 signature APOBEC mutations to predict G-to-A hypermutation. This additional analysis involved comparing the number of signature APOBEC mutations in a sequence to the results of the Los Alamos National Laboratories (LANL) HIV Sequence Database Hypermut2 program.

To compare the signature APOBEC mutation approach with the LANL Hypermut2 program, we analyzed HIV-1 *pol* plasma and peripheral blood mononuclear cell (PBMC) dideoxynucleoside direct PCR (Sanger) sequences in HIVDB encompassing either PR+RT, PR+RT+IN, or IN alone. The analysis included 211,400 sequences from plasma virus samples and 7,069 sequences from peripheral blood mononuclear cell (PBMC) samples. For each sequence, we determined (i) number of signature APOBEC mutations; (ii) the number of stop codons and active site mutations (i.e., PR D25N, RT D110N, D185N, D186N, and IN D64N, D116N, and E152K) resulting from G-to-A hypermutation; and (iii) the Hypermut2 rate ratio and its associated Fisher’s exact p value.

The accompanying table shows that for sequences containing <3 signature APOBEC mutations, 217,744 (99.97%) had a Hypermut2 rate ratio p value >0.05 (consistent with the absence of G-to-A hypermutation) and 62 (0.03%) had a Hypermut2 rate ratio p value ≤0.05 (consistent with the presence of G-to-A hypermutation). For sequences containing ≥3 signature APOBEC mutations, 212 (31.98%) had a Hypermut2 rate ratio p value ≤0.05 and 451 (68.02%) had a Hypermut2 rate ratio >0.05. Of the 62 sequences considered to be hypermutated by Hypmut2 but not by the signature APOBEC mutation approach, only 2 sequences (3.2%) had one or more stop codons or active site mutations. Of the 451 sequences considered to be hypermutated by the signature APOBEC mutation approach, 288 (63.9%) had one or more stop codons or active site mutations.

|  | | **LANL Hypermut2** | |
| --- | --- | --- | --- |
|  |  | **p ≤ 0.05 ⊕** | **p > 0.05 ⊖** |
| **Signature APOBEC mutation approach** | **n ≥ 3 ⊕** | 212 stop codons / active site mutations: 189 (89.2%) | 451 stop codons / active site mutations: 288 (63.9%) |
|  | **n < 3 ⊖** | 62 stop codons / active site mutations: 2 (3.2%) | 217744 stop codons / active site mutations: 5475 (2.5%) |
