# Supplementary figures and images for: Analysis of unusual and signature APOBEC-mutations in HIV-1 pol next-generation sequences
Source: PLoS One. 2020 Feb 26;15(2):e0225352. doi: 10.1371/journal.pone.0225352 (PMC7043932; doi:10.1371/journal.pone.0225352)

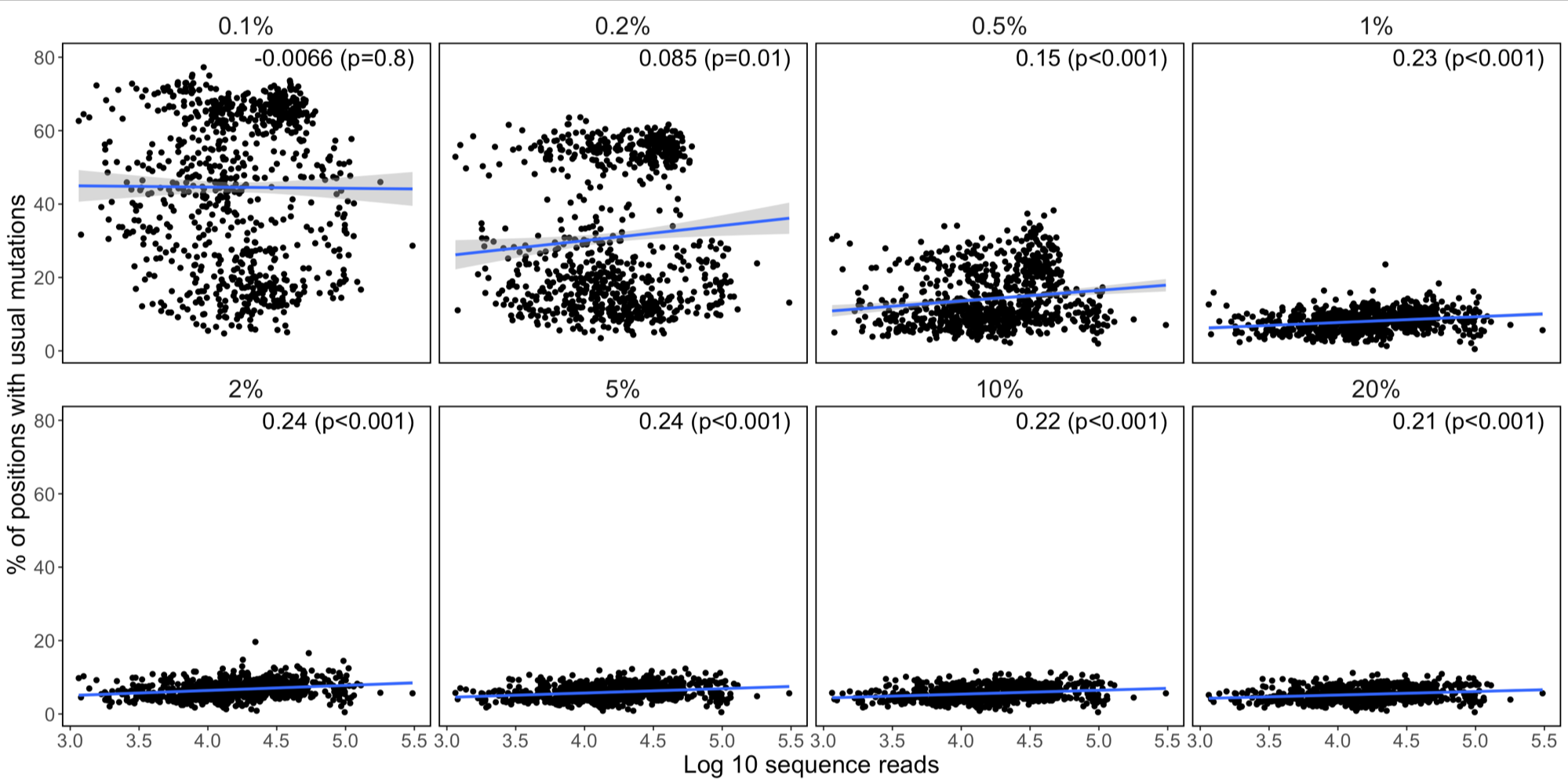

Supplement: S1 Fig — Each plot contains the Pearson correlation coefficient (r) and its associated p value. (TIFF) [file pone.0225352.s002.tiff]

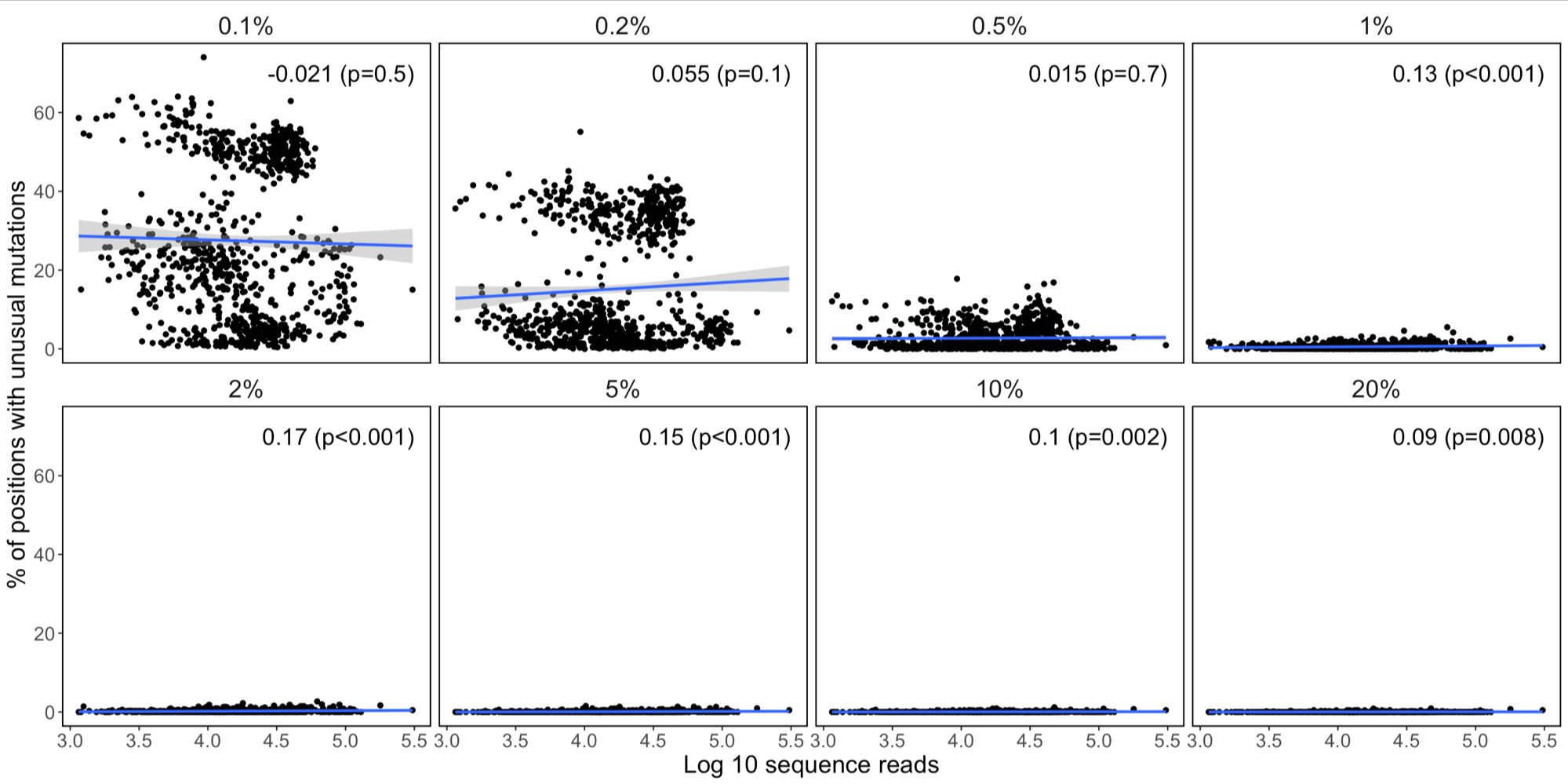

Supplement: S2 Fig — Each plot contains the Pearson correlation coefficient (r) and its associated p value. (TIFF) [file pone.0225352.s003.tiff]

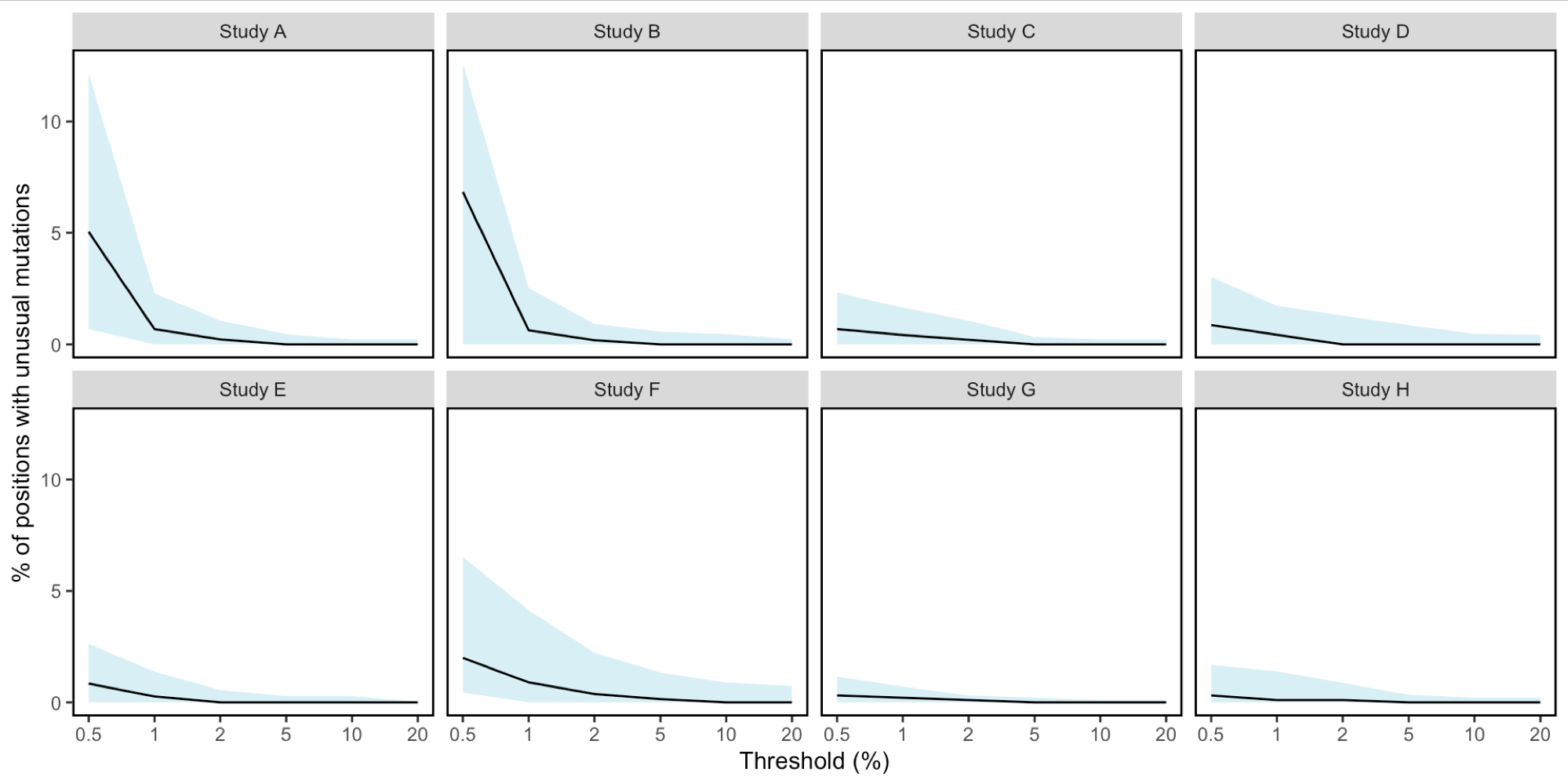

Supplement: S3 Fig — (TIFF) [file pone.0225352.s004.tiff]

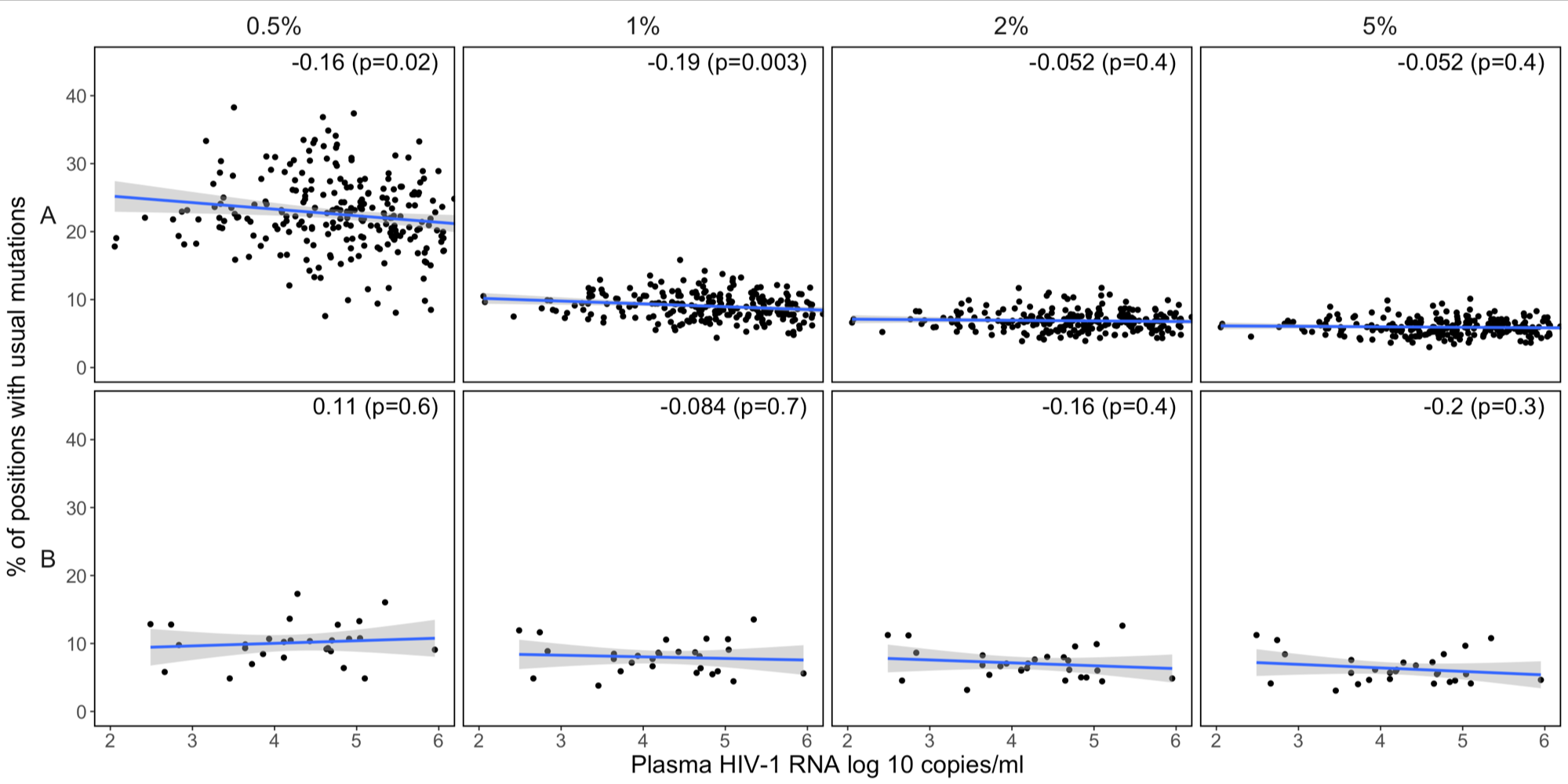

Supplement: S4 Fig — (TIFF) [file pone.0225352.s005.tiff]
